# A novel method for internal fixation of basal fifth metatarsal fracture in athletes: a cadaveric study of the F.E.R.I. technique (Fifth metatarsal, Extra-portal, Rigid, Innovative)

**DOI:** 10.1186/s40634-019-0213-5

**Published:** 2019-11-11

**Authors:** Pieter D’Hooghe, Silvio Caravelli, Simone Massimi, James Calder, Peter Dzendrowskyj, Stefano Zaffagnini

**Affiliations:** 1 Department of Orthopaedic Surgery and Sports Medicine, Aspetar Hospital, Doha, Qatar; 2 2nd Clinic of Ortopaedics and Traumatology, IRCCS Istituto Ortopedico Rizzoli, Bologna, Italy; 3 Department of Orthopaedic Surgery, Fortius Clinic, London, UK; 4 Department of Bioengineering, Imperial College London, London, UK

**Keywords:** Fifth metatarsal, Fracture, Internal fixation, Athlete

## Abstract

**Purpose:**

One of the main problems of Kirschner wire fixation of fifth metatarsal base fractures (in combination with a tension band wiring technique) seems to be hardware intolerance and several studies in athletes also report failure after isolated fixation with a screw only. These reports prompted us to look at new materials and a novel technique through fixation with an intramedullary screw combined with a high-resistance suture via the presented F.E.R.I. (Fifth metatarsal, Extra-portal, Rigid, Innovative) technique.

**Methods:**

This cadaveric study describes F.E.R.I. technique. On a cadaver, through two mini portals, a full reduction and solid internal fixation with an intramedullary screw and suture cerclage with Fiberwire of a fifth metatarsal base fracture is achieved. In this article, the cadaveric study and proposed surgical technique are explained and illustrated step by step.

**Results:**

The presented internal fixation F.E.R.I. technique is indicated in acute proximal fractures, stress fractures or non-union of metatarsal 5 (Zone 2–3 by Lawrence and Botte) and it resulted feasible and stable during manual stress test. The authors intend to study this technique in the clinical setting in the near future.

**Conclusions:**

Fifth metatarsal base fractures gain specific interest when occurring in athletes. In this group of patients, internal fixation is often required to obtain a satisfactory outcome and time to return to play. The aim of the presented cadaveric study is to illustrate an innovative concept of internal fixation, named F.E.R.I.

## Introduction

Sir Robert Jones (Jones 1902), in 1902, first described fractures at the metaphyseal/diaphyseal junction of the fifth metatarsal (MT-5). These make up approximately 70% of all metatarsal fractures (Petrisor et al. 2006), both in the general population and also in athletes. In an interesting review, Spang RC et al (Spang et al. 2018) have found that the overall percentage of fifth metatarsal base fractures in NFL players was 3.2%. Fifth metatarsal fractures are an increasing problem, particularly in athletes. Surgical treatment strategies and outcomes are, therefore, of particular interest among those involved in their care.

Orendurff et al.(Orendurff et al. 2009) demonstrated that during sport activities, bending moments and peak pressure are considerable through the fifth metatarsal. They highlighted how different athletic gestures, such as jump-off, landing and cutting movements can produce high tensile stress on bone structures - a forceful push-off of acceleration generates a high load on the forefoot, particularly on the fifth metatarsal (Orendurff et al. 2009). This can lead to either acute fractures of the base of the fifth metatarsal or, during high periods of activity, to stress fractures.

Pre-disposing factors include: cavo-varus foot, prominence of the fifth metatarsus, high body mass index (BMI), and the use of narrow-width shoes (Jastifer et al. 2017; McBryde Jr. 2009). An indirect force secondary to foot plantar-flexion and inversion - transmitted to the MT-5 by the peroneus brevis and plantar fascia traction (Mayer et al. 2014; Glasgow and Naranja Jr 1997) - is the usual mechanism of injury leading to this proximal fracture. Increased forces during physical activity make it easy to understand how athletes, in particular are predisposed to this type of injury.

MT-5 fractures have been classified by Lawrence and Botte (Lawrence and Botte 1993) according to the anatomical area affected, and also the mechanism of injury, in Zone 1 (tuberosity area), Zone 2 (tuberosity – metaphyseal area) and Zone 3 (metaphyseal – diaphyseal area). Fractures in Zones 2 and 3 are commonly referred to as Jones’ fractures, due to their similarity in symptoms, treatment and possible complications. Zones 2 and 3 are predisposed to delayed healing due to a vascular watershed zone (Smith et al. 1992; Le and Anderson 2017) between the insertion of peroneus brevis and the diaphyseal blood supply. Fractures interrupt the vascular channels in this area, leading to hypo-vascularisation and difficulty in complete healing.

Fractures of the proximal MT-5 can be treated either conservatively or surgically; Josefsson (Josefsson et al. 1994) demonstrated good functional outcomes with non-operative treatment. However, in athletes, treatment is usually surgical, because of the long recovery time, the higher risk of non-union and high rates of re-fracture associated with conservative treatment (Clapper et al. 1995; Kavanaugh et al. 1978; Raikin et al. 2008).

Several studies, however, report failure after fixation with screw, in particular, in athletes. Wright R. et al. (Wright et al. 2000) found that failure seemed to be correlated with the dimension rather than the type of screw (cannulated, cancellous or malleolar screws). He reported a 5% failure rate – a combination of refracture and nonunion. Granata JD. et al. (Granata et al. 2015) reported a 7.3% failure rate in patients with primary surgical treatment - mainly due to refracture. Because of this, other fixation methods have been developed aiming for better primary stability and a lower rate of complications and refractures. Internal fixation by tension band wiring has been used with similar outcomes (Delee et al. 1983; Lee et al. 2011). There have been many publications regarding fractures of the base of fifth metatarsal, however disagreement and controversy remain around the most appropriate surgical treatment.

This report describes and discusses an innovative concept for surgical fixation via a combination of screw and suture cerclage of MT-5 fractures in athletes, named F.E.R.I. “*Fifth metatarsal, Extra-portal, Rigid, Innovative”.*

## Materials and methods

**Fig. 1:**
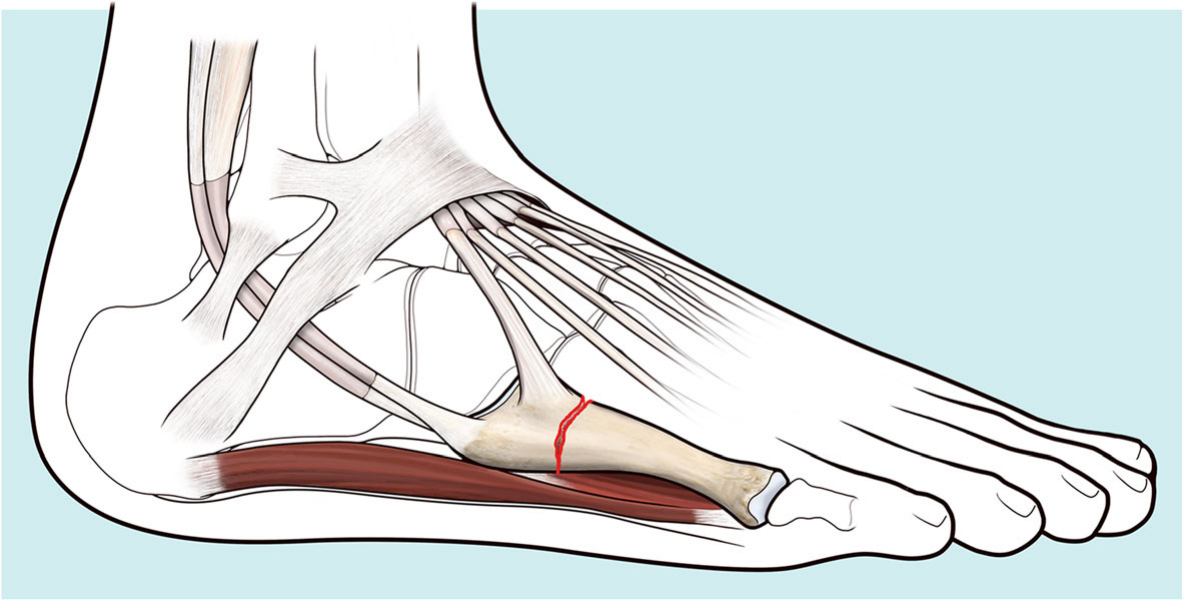
Proximal fractures or stress fractures of MT-5 (Zone 2–3 by Lawrence and Botte) can be treated with the F.E.R.I. technique

The “two portal technique” (TPT), or “F.E.R.I. technique”, is indicated for internal fixation of acute proximal fractures - or stress fractures of MT-5 - (Zone 2–3 by Lawrence and Botte) (Fig. 1). A further indication is chronic delayed union or non-union of proximal MT-5 fractures.

The feasibility of the technique has been tested on a cadaveric specimen. After creating a fifth metatarsal base fracture with a 10 mm osteotome, reduction and fixation has been performed under direct visual control.

### Surgical technique

A 1 cm longitudinal stab incision (“proximal portal”) is made 15 mm proximal to the base of the fifth metatarsal. This reveals the insertion of the peroneus brevis tendon. Dissection identifies the lateral dorsal cutaneous nerve and its distal branches.

**Fig. 2:**
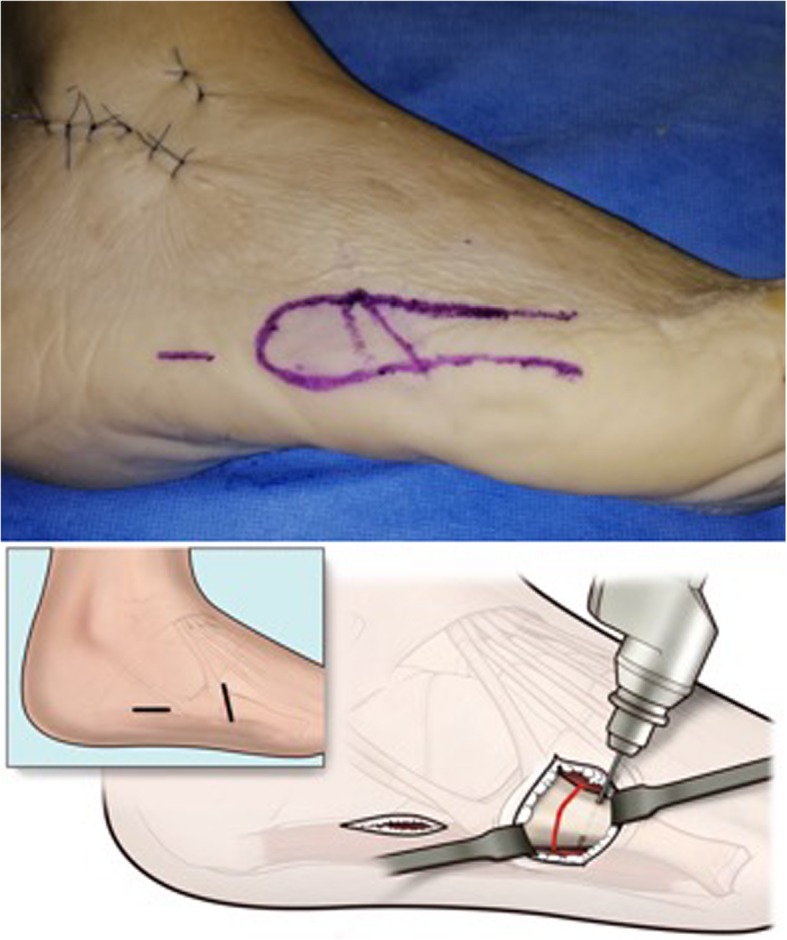
Creation of two portals allows for a minimally invasive technique, minimising soft tissue damage, when compared to classic tension band techniques or fixation with plate and screws

A second 15 mm incision (“distal portal”) is made obliquely, at the same level as the fracture site, following Langer’s skin folds, from dorsal to plantar and from proximal to distal (Fig. 2). Blunt subcutaneous dissection identifies the fracture line, which is then inspected and minimally debrided.

**Fig. 3:**
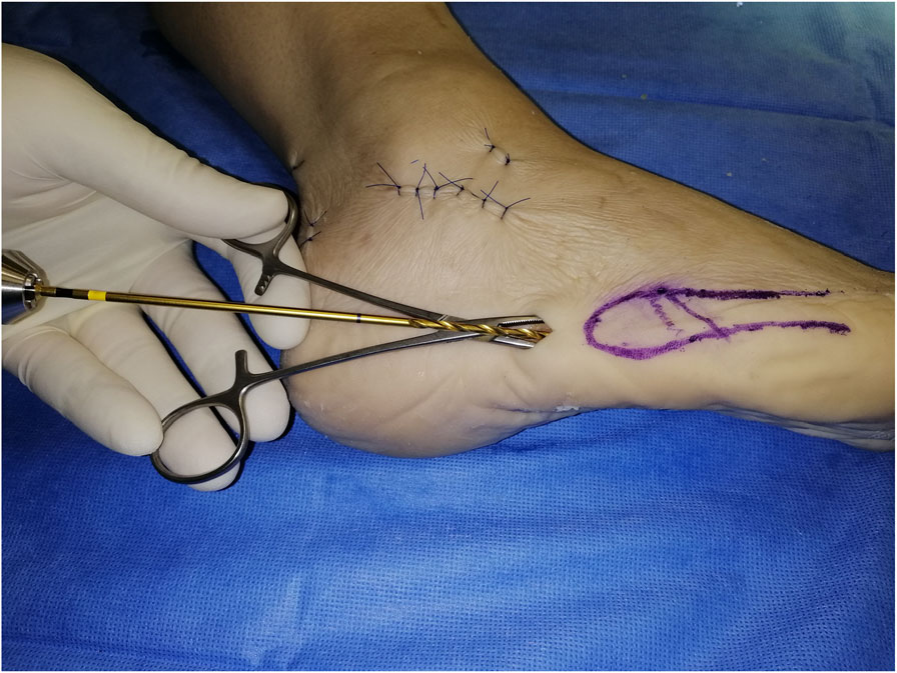
Preparation for screw fixation. A hole for the screw is drilled at the entry point. A 5.5 mm partially threaded screw is placed in the medullary cavity through the entry point and screwed to, or just beyond, the fracture line

**Fig. 4:**
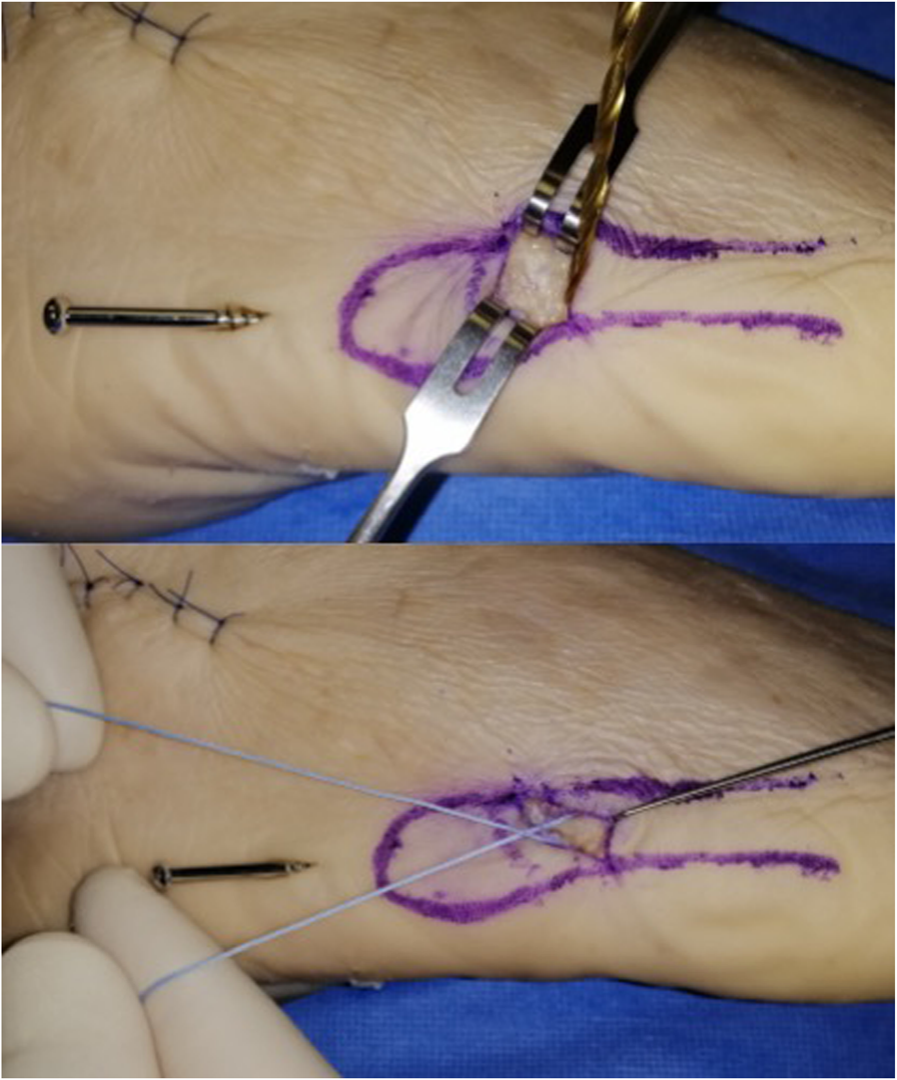
Preparation of cerclage. A through-hole is drilled vertically through the distal port, 10 mm distal to the fracture line. An Arthrex Fiberwire® (Arthrex, Naples, FL USA) no. 2 is passed through the hole using a figure-of-eight pattern

Under X-ray C-arm guidance, any displaced fracture is reduced. If a cannulated screw is used, a guide wire is then introduced through the proximal portal into the fifth metatarsal medullary cavity in a dorso-medial entry angle, medial to the tip of the base. This maximises screw length whilst protecting the insertion point of peroneus brevis (Fig. 3). A through-hole is drilled vertically through the distal port, 10 mm distal to the fracture line. An Arthrex Fiberwire® (Arthrex, Naples, FL USA) no. 2 is passed through the hole (Fig. 2 and Fig. 4).

**Fig. 5:**
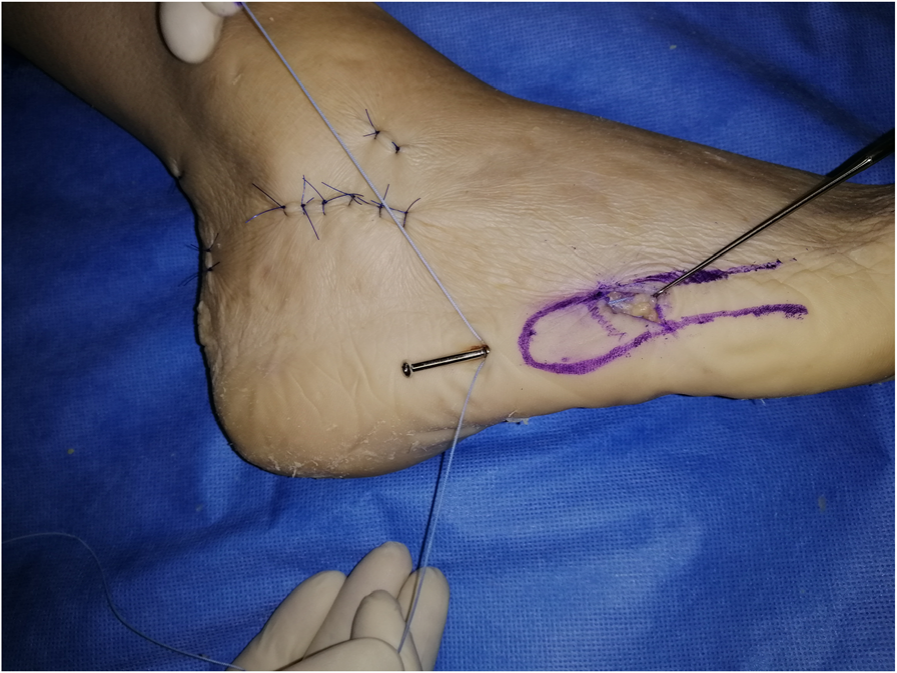
The Fiberwire® is crossed subcutaneously, using small curved Klemmer forceps, from distal to proximal in a figure-of-eight pattern and looped around the neck of the screw. The dorsal wire is pulled plantarly and the plantar wire dorsally

**Fig. 6:**
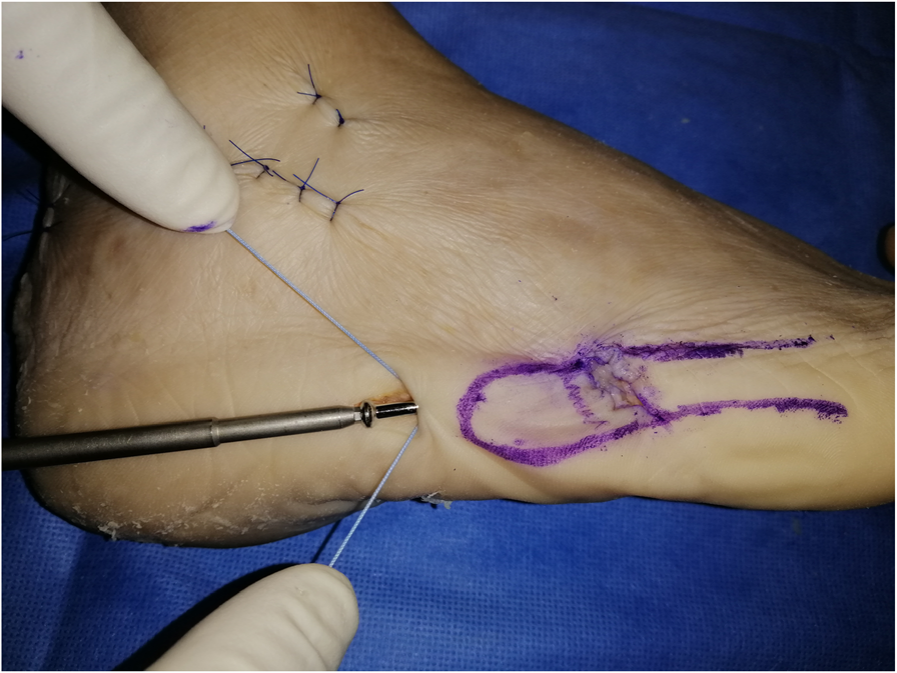
The screw is then tightened, the Fiberwire® cerclage is properly tensioned and securely knotted

**Fig. 7:**
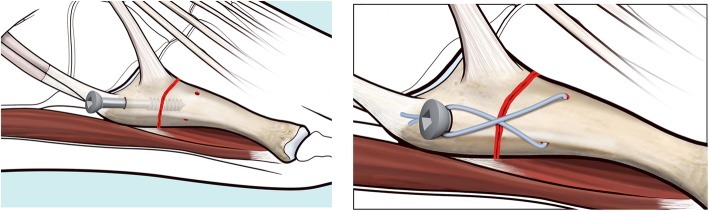
Diagram of screw position, of cerclage hole and final result. Note that the screw entry point in the figure has been deliberately drawn slightly lateral to aid visualisation

A 5.5 mm partially threaded screw is placed along the guide wire, if cannulated, and inserted into the medullary canal to across the fracture line (Fig. 3). After marking the dorsal end the suture wire with a marking pen, the Fiberwire® is then pulled subcutaneously from the distal incision to the proximal one in a figure-of-eight pattern and looped around the neck of the screw (Fig. 5). The screw is then tightened and the Fiberwire® cerclage properly tensioned and securely knotted (Fig. 6 and Fig. 7). Manual tests and maneuvers have been performed and fracture stability was achieved with success.

### Additional procedures

In cases of delayed union, or to improve fracture consolidation, a small amount of autogenous cancellous bone can be harvested from superolateral aspect of the calcaneus.

A 10 mm “extra-portal” is made posteriorily and slightly distal to the Sural nerve and peroneal tendon. Subcutaneous blunt dissection is performed to reveal periosteum, which is then reflected back with a periosteum elevator. The bone graft borders are visualised, and a carefully sized cortico-cancellous bone wedge can be harvested, using a small osteotome or a small oscillating saw.

Fluoroscopy can be used to identify the correct harvesting site. The cortico-cancellous autograft is placed like a wedge, with the cortical side positioned plantar, before suture cerclage and fracture compression. Positioning is aimed at the crossing point of the figure-of-eight cerclage, which can then support the wedge.

## Discussion

There have been many publications regarding fractures of the base of fifth metatarsal, however disagreement and controversy remain around the most appropriate surgical treatment.

Various techniques are available (Tsukada et al. 2012; Glasgow and Naranja Jr 1997; Wright et al. 2000) - the most popular being fixation by cannulated screw for proximal fractures of the fifth metatarsus (Kavanaugh et al. 1978; Lareau and Anderson 2015). Inserting a straight screw into the curved proximal fifth metatarsus can be a real challenge. The entry point for insertion needs to be immediately medial to the insertion fibres of peroneus brevis, avoiding branches of the Sural nerve (Donley et al. 1999; Fansa et al. 2012). Direction has to be 7° from cranial to caudal - relative to the sole of the foot - pointing in the direction of the diaphysis of the metatarsus.

Recently, several biomechanical studies have been carried out to evaluate the ideal characteristics of the screw to obtain a good fixation and grip (Duplantier et al. 2018; Huh et al. 2016; Scott et al. 2015; Nagao et al. 2012; Porter et al. 2009). The diameter of the screw should be no less than 4.5 mm in order for there to be adequate compression across the fracture line. In general, the wire should be between 4.5 and 5.5 mm in diameter- in men the diameter of the 5th metatarsus is greater than in women. There is no significant difference in the strength of fracture fixation between these different diameter screws (332.4 N versus 335.2 N between 4.5 and 5.5 screw) (Raikin et al. 2008).

Stainless steel cerclage is a type of internal fixation using the same concept as dynamic compression tension banding - tensile forces of bone eccentric loading are converted into compressive forces. Tension band fixation has undergone several modifications, since its introduction by Pauwels in 1980 (Pauwels 1980), ranging from the two-screws technique to the classical two Kirschner wires technique (Lee et al. 2011; Sarimo et al. 2006). Good results after tension band wiring fixation have been reported in the literature - it has become a well-documented procedure for the treatment of acute fifth metatarsal fractures and stress fractures (Sarimo et al. 2006; Nolte et al. 2016).

One of the main problems of fixation with Kirschner wires and tension band wiring, seems to be that of hardware intolerance, particularly in athletes. Several authors have reported the need to remove the orthopedic hardware due to this problem (Bryant et al. 2018; Curtis et al. 2015; Hasselman et al. 2003).

The classical technique involves two Kirschner wires inserted longitudinally in the metatarsal bone from the entry point at the medial base. Mofidi et al. (Mofidi et al. 2009), reported a case where a stress fracture of the fifth metatarsal was caused by tension band wiring itself - a known, though rare, complication. They describe a 26-year-old athlete who suffered a stress fracture 1 year after surgery, surmising that the loss of bone strength was due to both the presence in the medial cortex of the K-wire, as well as the hypo-vascularization of the bone segment.

These problems prompted us to look at new materials and novel techniques for the fixation of fractures at the base of the fifth metatarsal – particularly in athletes, who require greater stability for good functional recovery.

Stainless steel monofilament wire is strong and manageable and is still the material of choice for tension banding fixation in general traumatology practice. In recent years, many studies have compared other different synthetic suture materials (Flanigan et al. 2011; Mahar et al. 2006; Kuruvalli et al. 2008; Renner et al. 2014). A recent study by Westberg et al. (Westberg et al. 2017) compared the biomechanical characteristics of different sutures, including Fiberwire® (Arthrex, Naples, FL USA) and a classic 1.2 mm stainless steel monofilament, evaluating their cyclic load and load to failure. The authors highlighted that the Fiberwire® suture no. 2 had the highest tensile strength and load to failure of all materials in the study while the stainless steel monofilament had the lowest.

We suggest the use of both an intramedullary screw and a Fiberwire suture no. 2 cerclage together, combining the advantages of both techniques and reducing the possibility of failure. In patients with high functional demands, using an isolated intramedullary screw has been shown to be associated with poor resistance to dorsal flexion forces (Kavanaugh et al. 1978; Tsukada et al. 2012). This has resulted in either a greater risk of hardware rupture or insufficient stability of the fracture site. Stainless steel cerclage by itself can also fail if subjected to high load cycles, or can cause discomfort due to irritation of surrounding soft tissues.

The creation of two ports allows for this technique to be minimally invasive, useful for preserving the vascularity of the area, which is already somewhat hypovascularised. Minimal periosteal removal is likely to reduce the risk of delayed healing or non-union by protecting soft tissue coverage, when compared to other more aggressive internal fixation methods.

This technique has not yet been used clinically and is – at present - hypothetical in nature. Proof of concept has, however, been shown to be effective – it has been performed on a cadaveric specimen, illustrating surgical feasibility and technical reproducibility, but it has been not biomechanically tested and its superiority compared to standard techniques has to be still proved.

A second limitation of this technique is that it is not directed at the general population, but aimed at professional and elite athletes. This is because of their greater functional requirement and greater biomechanical stress on the fracture, with the possibility of failure of fixation if carried out with intramedullary screw only. Therefore, it makes sense to have a more stable synthesis, using a combination of the two techniques.

Fixation with an intramedullary screw combined with a Fiberwire suture no. 2 via the F.E.R.I. technique, is a valid treatment option for acute fractures and stress fractures of the fifth base in athletes. Furthermore, this technique could be used in cases of delayed consolidation or non-union in which a biological augmentation is requested. The authors intend to study this technique in the clinical setting in the near future.
